# Endodontic Indications among Patients Visiting a Tertiary Care Center: A Descriptive Cross-sectional Study

**DOI:** 10.31729/jnma.5424

**Published:** 2021-08-31

**Authors:** Deepa Kunwar, Archana Manandhar, Gita Gurung, Jwolan Khadka, Manisha Nepal

**Affiliations:** 1 Department of Conservative Dentistry and Endodontics, Gandaki Medical College, Pokhara, Nepal; 2 Department of Oral Medicine and Radiology, Gandaki Medical College, Pokhara, Nepal; 3 Department of Conservative Dentistry and Endodontics, Kist Medical College, Kathmandu, Nepal; 4 Department of Conservative Dentistry and Endodontics, Universal College of Medical Science, Bhairhawa, Nepal

**Keywords:** *endodontics*, *pulpitis*, *root canal treatment*

## Abstract

**Introduction:**

Endodontics is the study of prevention, diagnosis and treatment of diseases or injuries to the dental pulp. The ultimate goal of modern dental care is tooth preservation and root canal therapy/treatment is an available therapeutic strategy to retain teeth. The aim of the study is to find the prevalence of patients visiting a tertiary care center who had endodontic indications.

**Methods:**

The descriptive cross-sectional study included 516 patients accepted for endodontic treatment, between August 2019 and December 2019 in a tertiary care center. Ethical approval was taken from the ethical review board of Nepal Health Research Council (reference number: 4252019). Convenience sampling method was used. The data were entered in Statistical Package for Social Sciences version 20 software and analysed using descriptive statistics. Point estimate at 95% confidence interval was calculated along with frequency and proportion for binary data.

**Results:**

Out of 1740 patients, 516 (29.66%) (95% Confidence Interval= 21.46% - 27.51%) had endodontic indications. Symptomatic irreversible pulpitis 306 (59.30%) was the most prevalent pulpal disease. Maxillary teeth 300 (58.13%) had more endodontic diseases. While in individual dental elements most affected by endodontic diseases was the mandibular molar teeth 149 (28.87%). Females 348 (67.44%) were predominant for demanding endodontic management than males 168 (32.5%).

**Conclusions:**

More female patients and of younger age group in this study population demanded endodontic treatment. Irreversible pulpitis was responsible for the majority of the cases treated and more affected were the posterior teeth

## INTRODUCTION

Endodontic treatment is a judicious procedure of removing the infected dental pulp and periradicular lesions to preserve the inert nature of the tooth.^1^ Root canal therapy (RCT) is an easily available, efficacious therapeutic strategy to retain teeth.^2,3^ Loss of teeth affect not only basic mouth functions but aesthetic appearance and quality of life.^4,5^ Even for cancers or HIV-AIDS, endodontics management is preferred over extraction.^6^ There is increasing dental awareness and more people are desiring to keep their teeth.

Complications related to dental caries are the main reasons for prescribing endodontics treatment.^5,7^ Most frequently treated teeth are maxillary molars and premolars while the mandibular incisors are the least treated.^8^ Understanding indications and pattern of RCT will help in formulation of optimal preventive management policies.^9^

The aim of the study is to find the prevalence of patients visiting a tertiary care center with endodontic indications.

## METHODS

This descriptive cross-sectional study included all patients accepted for endodontic treatment, between August 2019 and December 2019 in the Department of Conservative Dentistry and Endodontics, Gandaki Medical College, Pokhara, Nepal after getting approval from the ethical review board of Nepal Health Research Council (reference number: 425-2019). The written and informed consent was obtained from all the patients. All patients above 15 years of age who attended Gandaki Medical College seeking endodontic treatment of their teeth were included in the study. Those patients who refused to give consent and patients who presented for re-treatment following failure of previous endodontic treatment were excluded from the study. Convenience sampling was done and the sample size was calculated by using standard formula,

n = Z^2^ × p × q / e^2^

  = (1.96)^2^ × (0.5) × (0.5) / (0.03)^2^

  = 1067

Where,

n = sample size,
Z = 1.96 at 95% Confidence Interval (CI),
p = prevalence taken as 50% for maximum sample size,
q = 1-p,
e = margin of error, 3%

However, we included 1740 patients in the study. Convenience sampling method was used to collect samples.

The need for endodontic treatment was confirmed by clinical assessment, vitality test and periapical radiograph.

All statistical analyses were performed using Excel spreadsheets and Statistical Package for the Social Sciences (SPSS) for Windows (Version 20). Descriptive analysis and point estimate for frequency and proportions were calculated.

## RESULTS

Out of 1740 patients, 516 (29.66%) (95% Confidence Interval = 21.46% - 27.51%) required endodontic indications in Gandaki Medical College, Pokhara, Nepal during the study period. Among endodontic indicated patient pulp diseases was prevalent in 306 (59.30%) of which symptomatic irreversible pulpitis 171 (41.08%) was the most prevalent. Chronic apical periodontitis 167 (44.18%) was the most prevalent periradicular disease (Table 1).

**Table 1:** Treated teeth due to pulp and periradicular pathologies according to gender.

Pulp	Male n (%)	Female n (%)	Total n (%)
Reversible pulpities	3 (0.58)	8 (1.55)	11 (3.1)
Symptomatic irreversible pulpities	65 (12.6)	106 (20.54)	171 (41.08)
Asymptomatic irreversible pulpities	21 (4.07)	21 (4.07)	42 (8.14)
Pulp necrosis	35 (6.78)	22 (4.26)	57 (8.52)
Internal resorption	1 (0.19)	0 (0)	1 (0)
External root resorption	2 (0.39)	3 (0.58)	5 (1.16)
Intensional Periradicular	13 (2.52)	6 (1.16)	19 (2.32)
Acute apical periodontitis	2 (0.39)	6 (1.16)	8 (2.32)
Chronic apical periodontitis	53 (10.27)	114 (22.09)	167 (44.18)
Acute apical abscess	2 (0.39)	5 (0.97)	7 (1.94)
Chronic apical abscess	8 (1.55)	19 (3.68)	27 (7.36)
Phonex abscess	0 (0)	1 (0.19)	1 (0.19)

Maxillary teeth were more prone to endodontic diseases than the mandibular teeth. The dental elements most affected by endodontic diseases in the maxilla were the incisors 78 (26%) while third molar 3 (1) being least affected (Table 2).

**Table 2:** Maxillary pathology (n=300).

Maxillary	n (%)
Incisor	78 (26)
Canine	19 (6.33)
First premolar	52 (17.33)
Second premolar	53 (17.66)
First molar	69 (23)
Second molar	26 (8.69)
Third molar	3 (1)

In the mandible, the first molars were the most affected 106 (49.07%) while canine 7 (3.24) were least affected (Table 3).

**Table 3:** Mandibular pathology (n=216).

Mandibular	n (%)
Incisor	12 (5.55)
Canine	7 (3.24)
First premolar	19 (8.79)
Second premolar	29 (13.42)
First molar	106 (49.07)
Second molar	35 (16.20)
Third molar	8 (3.70)

Endodontic management was more demanded by the female 348 (67.44%) than the male 168 (32.5%).

In the age group distribution, patients of age group 25-35 years 203 (39.34%) needed more endodontic management then other age groups. Age group of 5565 years 21 (4.06%) had least indication for endodontic management (Table 4).

**Table 4:** Age-group wise distribution of patients who received endodontic treatment.

Age group (years)	n (%)
15-25	99 (20)
25-35	203 (39.34)
35-45	156 (30)
45-55	37 (7)
55-65	21 (4.06)

## DISCUSSION

In this study, the demand for endodontic treatment was higher in females as they are more concerned about their oral health; hence they appeared to be better motivated to demand for oral health care. This correlate with previous reports.^8^

The need for endodontics was observed to be more in the age group of 25-35 years with 39% (n=203) with least demand in the age group 55-65 years. This result is in agreement with Umanah et al.^3^ who reported the highest number of root canal treatment among patients aged 21-29 years. This can be attributed to the high prevalence of dental caries and its complications reported in young adults. Study by Augusto et al. revealed a higher prevalence in the 46-60-year-old range (n= 8,460; 47.6%) and a decrease in subjects older than 60 years (n= 2,680; 20.6%).^10^

The result shows that the most prevalent diseases that demand endodontic management is symptomatic irreversible pulpitis in both males and females in pulpal diseases whereas chronic apical periodontitis is the most that demands endodontic management in periradicular diseases. The symptomatic irreversible pulpitis has been most prevalent for endodontic management, it may be due to unawareness of patient about the dental education and seek for the treatment only after the condition is symptomatic when they have pain or at advanced stage of dental diseases i.e. complicated dental caries and periodontal diseases. Incidentally, these major oral diseases are largely preventable. In our study, symptomatic irreversible pulpitis was the most prevalent one followed by chronic apical periodontitis, pulp necrosis, asymptomatic irreversible pulpitis, chronic apical abscess. This finding of symptomatic irreversible pulpitis has been boon by the study done by Umanah et al. having it as the most prevelant of all.^3^ But, the study by de Oliveira et al.had different finding, their study showed apical periodontitis as the most prevalent one for endodontic management.^9^ Similar, study by Ibhawoh LO, the most prevalent diagnosis made in was acute apical periodontitis followed by irreversible pulpitis.^11^ There is very least case of internal resorption, external resorption, phoenix abscess, it may be due to more trend of extraction of as soon as patient is symptomatic. The results of this study showed that the pulp diseases were detected in men and women in an unequal percentage may be because women take more care of their health and appearance than men. Similar were the findings by de Oliveira et al. where pulp diseases were detected in men and women in an unequal proportion the symptomatic irreversible pulpitis affected more females.^9^ The prevalence of periradicular disease was also unequal among males and females in our study but de Oliveira et al. on the other hand, men and women presented a similar prevalence of periradicular diseases.^9^

In accordance with our results, the literature shows that the posterior teeth are more often in need of endodontic treatment, anterior teeth have been found to be affected in 28% to 47% meanwhile posterior teeth have a frequency range of 53% to 72%.^12^ The maxillary arch had a higher proportion of teeth receiving endodontic treatment, a finding comparable to previous reports.^5,11^ Hence it is not surprising to find that the molar teeth accounted for majority of the teeth that received root canal treatment in this study, followed by the premolars and then the anterior teeth. This has been ascribed to their early eruption with subsequent higher susceptibility to dental caries, their morphology facilitates dental plaque accumulation and show large pulpal chambers while mandibular central and lateral incisors being the least susceptible teeth for caries.^11,13^ For the individual teeth that are indicated for endodontic management, the mandibular molars were the most frequently followed by the maxillary premolars in this study, a pattern similar to previous studies.^8,9^ Our study is in agreement with Wayman, et al. who reported in a survey covering a period of 8 years and 3350 consecutive endodontic patients, the most frequently treated tooth was the mandibular first molar, at 18% of the time.^12^ Umanah et al. (2012) observed that maxillary incisors were the most frequently treated teeth endodontically, followed by mandibular molars.^3^

Pain (n= 464; 90%) was the most common symptom recorded in this study. This is similar to the trend in developing countries with patients mostly seeking care when symptoms manifest.^14^ Discoloration and unaesthetic appearance were major reasons for seeking treatment predominantly for the anterior teeth.

This was a single-hospital study done in a limited sample size, and the patient characteristics of the study group may be different from other institutions.

## CONCLUSIONS

More female patients and younger age groups in this study demanded endodontic treatment. Irreversible pulpitis was the most prevalent diagnosed disease. The molars were the most endodontically treated teeth. Highest number of procedures was performed on maxillary teeth.
